# Use of Prazosin for Pediatric Post-Traumatic Stress Disorder With Nightmares and/or Sleep Disorder: Case Series of 18 Patients Prospectively Assessed

**DOI:** 10.3389/fpsyt.2020.00724

**Published:** 2020-07-22

**Authors:** Vladimir Ferrafiat, Maryam Soleimani, Boris Chaumette, Audrey Martinez, Jean-Marc Guilé, Brooks Keeshin, Priscille Gerardin

**Affiliations:** ^1^ Child and Adolescent Psychiatric Unit, URHEA, CHSR Sotteville les Rouen, Rouen, France; ^2^ Child and Adolescent Psychiatric Department, CHU Charles Nicolle, Rouen, France; ^3^ Department of Neurology and Neurosurgery, Montreal Neurological Institute and Hospital, McGill University, Montreal, QC, Canada; ^4^ Child and Adolescent Psychiatry Services, Amiens University Hospital, Picardie Jules Verne University, Amiens, France; ^5^ Department of Psychiatry, McGill University, Montreal, QC, Canada; ^6^ INSERM U1105 Research Group for Analysis of the Multimodal Cerebral Function, University of Picardie–Jules Verne (UPJV), Amiens, France; ^7^ Department of Pediatrics, University of Utah, Salt Lake City, UT, United States

**Keywords:** post-traumatic stress disorder, youth, prazosin, nightmares, sleep disorder

## Abstract

**Objectives:**

Few studies have investigated pharmacologic treatment for pediatric post-traumatic stress disorder (PTSD). Prazosin, an alpha-1 adrenergic receptor antagonist, has been studied and demonstrated to be efficacious in an adult population for PTSD related sleep disturbances; however, in the pediatric population, data is limited to case reports and retrospective case series. This study prospectively assessed the safety and effects of Prazosin on PTSD symptoms in a pediatric sample.

**Methods:**

Since 2016, 18 patients with PSTD under the age of 15 admitted in a child and adolescent psychiatric unit were challenged with prazosin as part of a treatment protocol. PTSD symptoms and adverse effects were collected weekly and prospectively assessed each month with validated clinical scales. All data were retrospectively analyzed. This treatment protocol and the evaluation of clinical data were approved by our Ethical committee for research on preexisting data at the University Teaching Hospital of Rouen.

**Results:**

Among the 18 patients (10 girls and 8 boys), 13 (72%) had experienced sexual abuse and 5 (28%) family violence. After 1 month of treatment with a mean prazosin dose of 2.16 ( ± 0.6) mg/day, the CGI-S score significantly decreased from 5.3 ( ± 0.9) to 2.9 ( ± 0.7) (improvement of 43%). The mean total UCLA-PTSD-RI score significantly decreased 11.4 points ( ± 5.4) during the first week and 37.9 ( ± 16) during the first month, leading to an improvement of 20% and 67%, respectively. The improvement was significant irrespective of trauma exposure or sex. No adverse effects were reported except for one patient (hypotension).

**Conclusion:**

Consistent with prior case reports and retrospective reviews, our retrospective analysis of data prospectively and systematically assessed among 18 patients suggests that prazosin is well-tolerated and associated with improvement in symptoms for pediatric PTSD.

## Introduction

Patients with post-traumatic stress disorder (PTSD) persistently re-experience events with intrusive symptoms, such as intrusive thoughts, nightmares, and flashbacks. Additionally, avoidance of trauma-related stimuli, negative alterations of cognition and mood, and alterations in arousal and reactivity are required to meet criteria of PTSD (1). Children may re-enact traumatic or rescue scenes rather than describe flashbacks. Adolescents may deal with intrusive symptoms through body image preoccupations, injuries, and risky behaviors (2). Many factors impact the clinical features, such as age, gender, type of trauma, and possibility of a protective support (3).

The prevalence of pediatric PTSD fluctuates depending on the population studied. Some studies reported a prevalence around 0.11% of children aged 7 to 9 (4) and 0.5% of children aged 9 to 16 (5). Another study estimated 3%–6% (6), which is more consistent with adult studies. There is a gender difference with a higher rate of PTSD in females (7) and violent and sexual traumas are associated with higher rates of PTSD (5). Multiple and/or chronic exposures may have a cumulative effect on the severity and type of symptoms (8). Sleep disturbances and nightmares independently affect the severity and course of the disease (9), whereas cognitive impairments are associated with exposure to trauma, resulting in the development of learning challenges. Persistence of PTSD symptoms can result in psychological sequelae such as comorbid psychiatric disorders (anxiety disorders, depression) and suicide (8).

To date, trauma-informed psychotherapies are the first-line treatment for pediatric PTSD. The type of treatment may be influenced by prior experience of the clinician and the availability of treatments, with minimal application of evidence-based recommendations (10). Although trauma-informed psychotherapies have the greatest evidence of efficacy in symptom reduction and remission (11), access is limited in many countries. The use of pharmacologic treatments in youth is based on data from adult populations. Only a few studies have prospectively and systematically evaluated the use of pharmacological agents in youth with PTSD. One open-label study with citalopram (12) and one double-blind, placebo-controlled 12-week study with a fixed dose of fluoxetine (13) demonstrated improved symptoms over the course of treatment; however, randomized controlled trials of antidepressants, both tricyclic and serotoninergic, have not shown benefit (14–16). Similarly, second-generation antipsychotics (SGA) may be associated with improvement in some PTSD symptoms, but these results are supported by very few pharmacological studies, limited to case series (17, 18) and open-label studies for risperidone (19) and quetiapine (20). Regarding antiepileptic agents, carbamazepine was associated with improvement in PTSD symptoms including nightmares in a series of pediatric patients with sexual abuse-related PTSD (21), and divalproex was associated with improvement in boys with PTSD and comorbid conduct disorder (22).

Recently, studies on the pathophysiology and neuroendocrine mechanisms of PTSD suggest the hyperactivation of the catecholaminergic system may be associated with PTSD symptoms (23–25). Some literature has evaluated medications that reduce noradrenergic activity in youth suffering from PTSD. In an open trial, clonidine was associated with decreased hyperarousal symptoms (26) and in a case study, clonidine was associated with reduced reenacting symptoms (27). In a pilot study, propranolol was associated with re-experiencing and hyperarousal symptoms (28); however, in a prevention randomized controlled trial, propranolol showed no benefit for preventing pediatric PTSD at 6 weeks of follow-up (29).

Prazosin is an alpha-1 adrenergic receptor antagonist. Most (30–32), but not all (33) studies of prazosin in adults with PTSD have demonstrated its efficacy when treating PTSD associated nightmares and sleep disturbances. Several pediatric case reports with prazosin have been reported (34). In a recent retrospective chart review of 40 children and adolescents, Keeshin et al. demonstrated that prazosin is well-tolerated and associated with a significant improvement of nightmare frequency and dysomnias (35). In line with previous findings, we conducted a retrospective analysis of data prospectively and systematically assessed from youth with PTSD who received prazosin as part of a clinical protocol in our specialized unit. The aim of the study was to quantify the effects of prazosin on PTSD associated nightmares and sleep disturbance, closely and systematically monitoring for safety within a pediatric cohort.

## Materials and Methods

### Participants

All patients consecutively admitted to our child and adolescent psychiatric unit (University Unit, Hospital CH Rouvray) between January 2016 and December 2017 were systematically assessed for a diagnosis of PTSD. A total of 173 patients including all diagnoses were admitted within this period and 20 (11.6%) met criteria for PTSD.

All participants were children and adolescents younger than 15 years old. The diagnosis of PTSD was determined using the DSM-5 criteria through two clinical interviews conducted separately by the two senior clinicians (VF and MS) and measured using “The University of California at Los Angeles Post-traumatic Stress Disorder Reaction Index” (UCLA-PSTD-RI). A cut-off for the UCLA-PSTD-RI was fixed to ≥38 (36). The diagnosis of PTSD with severe nightmares and/or sleep disorder was confirmed by pooling interview data from the two senior clinicians and self-report UCLA-RI data. To be confirmed, each interview had to meet DSM-5 criteria for PSTD and UCLA-PSTD-RI score had to be equal or above the cut off.

Inclusion criteria: all patients with a diagnosis of pediatric PTSD presenting with nightmares and/or sleep disorders, regardless of the previous pharmacological treatments. Other psychiatric conditions or comorbidities among this group included major depressive disorders (MDDs), bipolar disorder, other mood disorders, anxiety disorders, obsessive compulsive disorder (OCD), mild-moderate ASD, mild-moderate intellectual disability (DI), TICS and Tourette syndrome, ADHD, DMDD, learning disabilities. All treatments for others comorbid psychiatric conditions were maintained during the admission, if they were considered necessary. No patients received trauma-focused psychotherapy until PSTD symptoms were improved in order to not influence the effect of prazosin on PTSD symptoms.

Exclusion criteria were defined as follows: (i) age less than 6, as the UCLA-PSTD-RI is invalid for children under 6 years old; (ii) any history of cardiovascular disorders which may contraindicate the use of prazosin or any history of previous adverse events with prazosin; (iii) refusal to be evaluated by the clinical scales (opposition with refusal to respond, language difficulties, unreliable answers); (iv) severe or profound intellectual disability, severe autism spectrum disorder (ASD), schizophrenia and other chronic psychosis.

Once the diagnosis of PTSD with severe nightmares and/or sleep disorder was confirmed as detailed above, all patients who met criteria were systematically and prospectively assessed and treated with off label prazosin as part of our clinical protocol. This process, using standardized clinical data as part of our practice is routine, allowing for clinical assessment of any improvement through validated clinical scales. Every admitted patient goes through this process, regardless of his/her psychiatric disorder. This procedure also stands as our standard process for any off-label use of treatment in our admitted patient. All standardized data were prospectively recorded through the patient’s medical file. No sensitive data were recorded and only data for clinical purpose were retrospectively used and analyzed anonymously.

All families provided informed consent to the use of prazosin for their children, after being fully informed of: i) the off-label use and its scientific rationale in the matter of treating PTSD intrusive symptoms; ii) the pharmacokinetics of prazosin; iii) the specific symptomatic targets aimed with prazosin; iv) possible side effects and contraindications known with prazosin; and v) the specific side effect monitoring schedule. No family or legal guardian refused the offer of prazosin treatment. This protocol E2020-17 was approved by our Ethical committee for research on preexisting data at the University Teaching Hospital of Rouen. The committee concluded that this study do not present any ethical issue and any violation of “*Loi n° 2012-300 du 5 mars 2012 (dite loi Jardé)*”. This study is consistent with and conforms to French Law regarding clinical research.

### Clinical Assessment

During the first week of admission, the assessment included the following: (1) sociodemographic data (age, sex, country of origin, and family socioeconomic status); (2) a semi-structured interview Kiddie-Schedule for Affective Disorders and Schizophrenia-Present and Lifetime (K-SADS-PL) to evaluate for personal and family histories of psychiatric and medical disorders, including axis-1 comorbidities; (3) a clinical examination; (4) the administration of the UCLA-PSTD-RI to confirm and quantify the severity of PTSD; (5) the sleep score calculated from the ULCA-PTSD-RI to specifically evaluate the quality and degree of sleep disturbance; and (6) the Clinical Global Impression-Severity scale (CGI-S). Each scale was scored at the baseline, every week during hospitalization and at discharge.

The validity and psychometric properties of the DSM-5 version of the UCLA-PTSD-RI have been validated in the pediatric population (37). The UCLA-PTSD-RI is a self-report questionnaire that assesses trauma history and diagnostic criteria for PTSD based on frequency of symptoms. Symptoms are reported in five clusters: (1) intrusive symptoms such as unwanted upsetting memories, flashbacks, nightmares (cluster B); (2) avoidant symptoms such as avoidance of thoughts, feelings, or reminders (cluster C); (3) negative cognition/mood symptoms such as inability to recall the trauma, negative affect and thoughts, culpability, decreased interest (cluster D); (4) hyperarousal symptoms such as irritability, angry outbursts, risky behavior, hypervigilance (cluster E); and (5) dissociative symptoms such as depersonalization, derealization (cluster A). The scale has been validated for children over 6 years old (38). A cut-off was fixed at ≥38. This cut-off showed the greatest sensitivity and specificity for detecting PTSD (36).

The sleep score is an added subscale to the UCLA-PTSD-RI to monitor for sleep problems, summing questions 10 and 21 from the UCLA-PTSD-RI, with a score range of 0 to 8.

The CGI provides a brief assessment of the clinician’s view of the patient’s global functioning prior to and after initiating a treatment (39). The CGI-S is a 7-point scale that requires the clinician to rate the severity of the patient’s illness at the time of assessment: 1 = Normal, not at all ill; 2 = Borderline mentally ill; 3 = Mildly ill; 4 = Moderately ill; 5 = Markedly ill; 6 = Severely ill; 7 = Among the most extremely ill patients.

All assessments except UCLA-PTSD- RI (self-questionnaire) were performed and reviewed by senior child and adolescent psychiatrists (MS and VF) with expertise in pediatric PSTD.

### Treatment Introduction, Maintenance, and Response

Prazosin was initiated at 1 mg per day at bedtime, consistent with previous studies (40). The dosage was then increased by 1 mg every week until clinical improvement, as long as tolerance was acceptable (35). No other treatments targeting PSTD features besides prazosin were introduced during the trial. We defined the response to treatment by a reduction ≥25% of the UCLA-PSTD-RI global score in accordance with other reports (41) and the remission of PTSD could be considered from a score less than 38 (UCLA-PTSD index cut off) (36). As prazosin has been reported to have a significant effect on symptoms during the first weeks in adults, the duration of the study was 4 weeks. For clinical purpose, maintenance treatment was proposed to patients at the end of the study in cases of significant improvement. Most of our admitted patients were admitted while taking others agents such as antidepressants, antipsychotics and psychostimulants for comorbid psychiatric disorders (MDD, anxiety disorders, ADHD) or symptoms of PTSD. As part of the treatment protocol, we maintained treatments without changing dosage at the time of initiating prazosin and during prazosin dosage adjustment.

Common side effects associated with prazosin therapy include: i) dizziness, drowsiness, headache, faintness, and syncope for the nervous system; ii), palpitations, hypotension (reported as rare) for the cardiac-vascular system; iii) constipation, diarrhea, dry mouth, nausea, vomiting for the gastrointestinal system; iv) urinary frequency for the renal and urinary system; and v) reduced response to environmental allergens for the immune system. Blood pressure (BP) and heart rate were manually monitored, measured, and recorded on a daily basis twice a day during the first week of treatment and during the week after each dose increase. BP was manually measured in two positions, first in supine position and after in seated still position in a quite nursing room, in order to capture orthostatic hypotension. Heart rate was manually measured following seated BP. After initial close monitoring, BP, and heart rate were manually measured once a week in the morning until the end of the hospitalization. All other known adverse events (headache, enuresis, hypotension, …) were systematically evaluated and, when present, monitored by senior physicians through a checklist (part of the medical file) on a weekly basis through a general clinical work up and a full side effect clinical interview. Any new side effect that could be imputable to prazosin was recorded and reported to pharmacology-toxicology department, as our standard procedure requires.

### Statistical Analyses

Statistical analyses were conducted using SPSS software (IBM SPSS Statistics for Windows, Version 24, IBM Corp., Armonk, NY). Kolmogorov Smirnov tests identified that changes in the UCLA and sleep scores did not follow a normal distribution. Due to the small number of participants and the impossibility to assume a normal distribution, non-parametric tests were used. Differences in the mean of the clinical scores were compared to zero (null hypothesis) using a Wilcoxon’s test. Comparisons between two groups were tested using a Fisher’s exact test and tests between more than two groups were examined with a Kruskal-Wallis’s test. Correlations were tested using both Pearson’s and Spearman’s tests. A bilateral alpha-risk of 0.05 was assumed. Graphical representations were done using SPSS.

## Results

### Description of the Population

In total, 18 patients entered the treatment protocol and received prazosin: 10 girls and 8 boys ages 9 to 15. Two types of trauma were present: sexual abuse (rape, sexual touching) and family violence (physical violence, psychological violence). Sexual abuse was the most common type of trauma, present in 90% of the girls and 50% of the boys. Psychiatric comorbidities were present in almost half of the patients and most patients (83.1%) received a concomitant psychotropic medication either for the comorbidities (anxiety disorder, MDD) or previously identified PTSD. Details of the sociodemographic and clinical characteristics are shown in **Table 1**.

**Table 1:** Sociodemographic and clinical characteristics of the patients.

Age (years ± standard deviation)	9 to 15 (13 ± 1.8)
**Gender** (number and percentage)	
Male	8 (44.4%)
Female	10 (55.6%)
**Ethnicity** (number and percentage)	
Caucasien	16 (88.8%)
African	2 (11.1%)
**Socioeconomic status** (number and percentage)	
Low	12 (66.6%)
Middle	6 (33.3%)
High	0 (0%)
**Type of trauma** (number and percentage)	
Sexual abuse, including:	13 (72.2%)
Single sexual abuse	4 (22.2%)
Repetitive sexual abuse	9 (50%)
Familial violence	5 (27.8%)
**Clinical description** (number and percentage)	
Comorbidities, including:	8 (44.4%)
MDD	7 (38.9%)
Anxiety disorder	1 (5.5%)
Patients receiving medication, including	15 (83.1%)
SSRI	5 (27.7%)
Carbamazepine	5 (27.7%)
AP	5 (27.7%)

MDD, major depressive disorder; SSRI, selective serotonine reuptake inhibitor; AP, antipsychotics.

### Intensity of Symptoms at Baseline

The mean scores for the full measure and subscales are reported in **Table 2**. The mean UCLA-PSTD-RI at baseline was 56.44 (SD ±12.784). The CGI-S score at baseline was 5.28 ( ± 0.9).

**Table 2:** Description of scores at baseline (mean ± standard deviation) and after 4 weeks of treatment by prazosin.

Variable	Baseline(mean ± standard deviation)	After four weeks(mean ± standard deviation)	Percentage of improvement (%)	Significance compared to no improvement
**UCLA-A**	2.89 ± 1.64	0.44 ± 0.62	84.6%	p < 0.001
**UCLA-B**	16.44 ± 3.60	2.61 ± 2.70	84.1%	p < 0.001
**UCLA-C**	4.89 ± 2.08	0.78 ± 0.94	84.1%	p < 0.001
**UCLA-D**	16.67 ± 5.41	7.39 ± 3.73	55.7%	p < 0.001
**UCLA-E**	18.67 ± 4.46	7.67 ± 4.39	58.9%	p < 0.001
**UCLA-Total**	56.44 ± 12.78	18.50 ± 9.65	67.2%	p < 0.001
**SLEEP score**	6.44 ± 1.62	1.33 ± 1.53	79.3%	p < 0.001
**CGI-S**	5.28 ± 0.90	2.94 ± 0.73	44.1%	p < 0.001

CGI-S, Clinical Global Impression-Severity; UCLA-A, cluster A of ULCA-PTSD-RI; UCLA-B, cluster B of ULCA-PTSD-RI; UCLA-C, cluster C of ULCA-PTSD-RI; UCLA-D, cluster D of ULCA-PTSD-RI; UCLA-E, cluster E of ULCA-PTSD-RI; UCLA-Total, total score of ULCA-PTSD-RI.

### Prazosin Dosage After 4 Weeks of Treatment

The final dose of prazosin ranged from 1 mg/day to 3 mg/day, with 11%, 61%, and 28% respectively receiving 1, 2, and 3 mg. The mean dose was 2.16 ( ± 0.6) mg/day. The final prazosin dose for each patient is available in the **Supplementary Table 2**.

### Improvement of ULCA-PTSD-RI Score After 4 Weeks of Treatment

Prazosin was associated with a significant improvement of PTSD symptoms, assessed by decreases in UCLA-PTSD-RI scores. The mean total UCLA-PSTD-RI score decreased by 37.9 ( ± 16) after 4 weeks, with a mean score improvement of 67%. The UCLA-PSTD-RI score improvement was more than 0% (p < 0.001) and 25% (p < 0.001) in all 18 patients. Improvement occurred in each subscale, with the most improvement noted among intrusive symptoms (cluster B), avoidance symptoms (cluster C) and dissociative symptoms (cluster A) (around 84% improvement noted in each subscale). In contrast, symptoms related to negative alterations in cognition and mood (cluster D) and hyperarousal (cluster E) were less responsive with improvement ranging between 55 and 59%.

### Improvement of Sleep Score After 4 Weeks of Treatment

Mean sleep score at baseline was 6.44 ( ± 1.62) and 1.33 ( ± 1.53) at week 4. Sleep scores were also significantly improved at week 1 and 4, with a mean difference of -1.38 (p < 0.001) and −5.94 (p < 0.001) respectively.

### Improvement of CGI-S After 4 Weeks of Treatment

CGI-S scores were also significantly improved during treatment with prazosin at 4 weeks.

**Figure 1:**
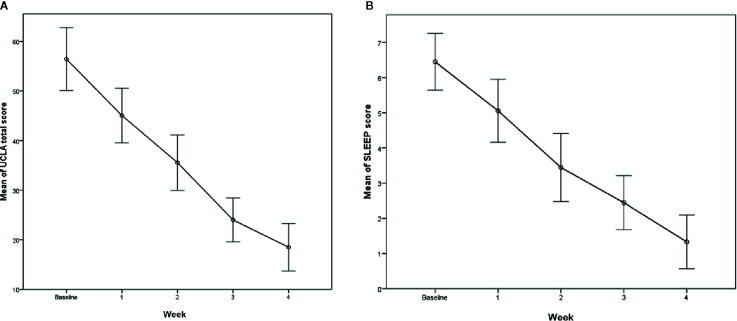
**(A)** Evolution of the UCLA-PSTD-RI score within 4 weeks. **(B)** Evolution of the Sleep score within 4 weeks.

**Figure 2:**
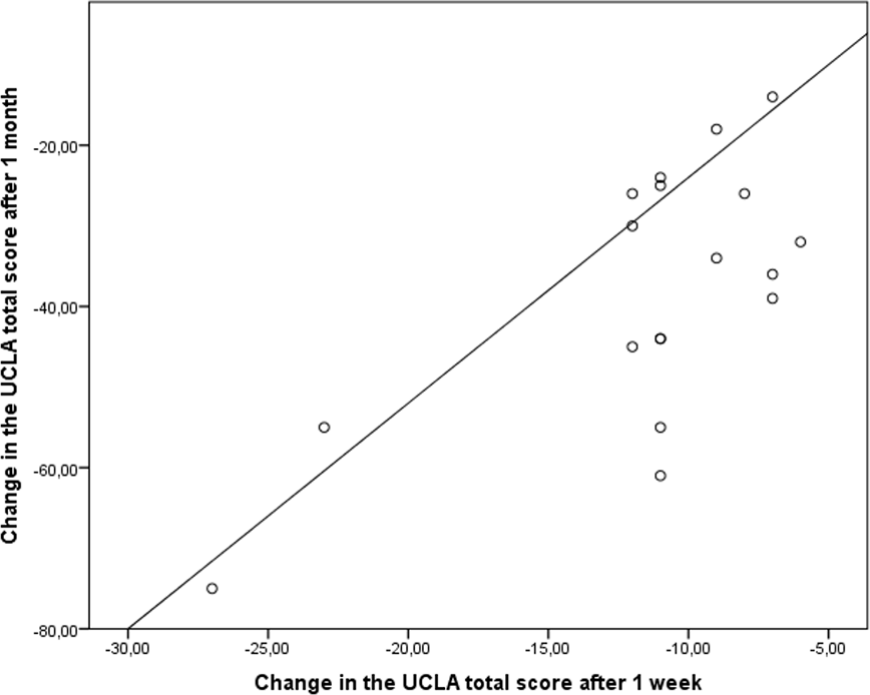
Correlation between clinical response to prasozin at week 1 and at week 4.

All the results are summarized in **Table 2** and **Figures 1A, B** and **2**.

### Early Response to Prazosin

The full UCLA-PSTD-RI score decreased 11.4 points ( ± 5.4) during the first week, with a mean score improvement of 20% (**Supplementary Table 1**). Improvement was significantly different from baseline with a dose of prazosin of 1 mg/day. Moreover, early response to treatment may be associated with a better response to treatment at 4 weeks (**Figure 2**). A potential correlation was found between week 1 and week 4 improvement for the full UCLA-PSTD-RI (Spearman’s test r = 0.45; p = 0.064). A significant positive correlation between improvement at week 1 and at week 4 for Sleep score (Spearman’s test r = 0.468; p = 0.05).

With regard to dose, patients receiving 2 or 3 mg of prazosin showed a greater improvement in CGI than the patients receiving 1 mg, but this distinction was not found in the UCLA PTSD RI. No differences were shown between patients receiving 2 and 3 mg.

### Tolerance

In general, prazosin was well-tolerated. The mean systolic BP remained stable, estimated at 111.94 mm Hg ( ± 10.5) at baseline and 110.88 mm Hg ( ± 8.3) after the 4 weeks. The same stability was noted for diastolic BP, with a mean of 72.44 mm Hg ( ± 11.2) at baseline and 73.16 mm Hg ( ± 7.8) at 4 weeks. The mean heart rate before and after treatment was 87.44 bpm ( ± 11.4) and 89.5 bpm ( ± 11.1), respectively. There were no treatment-related differences in between morning and evening BPs. The patient’s weight was monitored and did not differ before and after the treatment. The results are summarized in **Table 3** and full assessment is reported in **Supplementary Table 2**. One patient (Patient n°1) reported symptomatic hypotension with dizziness and tachycardia at week 2, following the increase of the dosage of prazosin at 2 mg. His morning BP was 64/46, whereas the night before it was noted to be 104/66, and heart rate increased to 130 bpm from a prior recording of 77 the prior evening. The patient was encouraged to increase hydration and prazosin was decreased to 1mg/d, with resolution of symptoms within 48 hours. Of note, this patient did not receive any other drugs associated with hypotension and the 1-mg dosage remained well-tolerated with no further hypotension.

**Table 3:** Description of tolerance variables at baseline (mean ± standard deviation) and after 4 weeks of treatment by prazosin.

Variable	Baseline Mean (± standard deviation)	After 4 weeks Mean (± standard deviation)
Systolic blood pressure (mmHg)	111.94 (± 10.5)	110.88 (± 8.3)
Diastolic blood pressure (mmHg)	72.44 (± 11.2)	73.16 (± 7.8)
Heart rate (bpm)	87.44 (± 11.4)	89.5 (± 11.1)
Weight (kg)	53.25 (± 13.1)	53.5 (± 13.4)

### Sub-Groups Analyses Based on Sex and Type of Trauma

Due to the small number of participants, the subgroup analyses were exploratory. Overall, baseline UCLA-PSTD-RI full measure and subscales were not different between groups based on type of trauma or sex (**Supplementary Table 3**). The decrease in UCLA-PSTD-RI after 4 weeks was significant for each type of trauma (p = 0.001 for sexual abuse; p = 0.043 for family violence) and both sexes (p = 0.012 for male; p = 0.005 for female).

## Discussion

To our knowledge, this is the first inpatient study with the largest sample of youth systematically and prospectively assessed with standardized and validated clinical scales on the efficacy and tolerance of prazosin for pediatric PTSD with nightmares and/or sleep disorder. We identified that prazosin is associated with significant improvement on the UCLA-PSTD-RI as well as within each subscale, especially intrusive symptoms and among Sleep associated symptoms. Prazosin was well tolerated with similar results in both sexes. Our results are consistent with the recent retrospective case series from Keeshin et al. (35) showing prazosin associated with improvement in nightmares and sleep disturbance.

Our study adds additional support for the use of prazosin among children and adolescents with PTSD. In the evaluated cohort, prazosin was associated with rapid improvement, as demonstrated with a significant improvement after only 4 weeks of treatment. Moreover, even a small dosage of prazosin (1 mg per day) was associated with a decrease of both symptom burden and functional impairment caused by PTSD. The positive correlation between improvement at week 1 and at week 4 for Sleep score suggests that if a patient starts to respond to prazosin on 1 mg, ongoing responsiveness at week 4 is possible. Given that many youth responded to doses of 2 mg or higher, our data support that clinical response is likely to be expected from and beyond 2 mg/day. Furthermore, the clinical improvement of our patients was not limited to nightmares and sleep disturbance. Indeed, prazosin was associated with improvement in every cluster of PTSD symptoms, including avoidance and negative alterations of cognition and mood.

By improving sleeping difficulties and physical manifestations of traumatic stress at night due to its action on the sympathetic system, prazosin may indirectly positively enhance mood regulation, arousal, and cognitive abilities such as attention and memory during the day. Moreover, a multidisciplinary and integrative approach to pediatric PTSD including nursing, psychotherapy (DBT, EDMR), treatment of psychiatric and developmental comorbidities, family therapy, and guidance for social and judicial procedure, may enhance this cycle of PTSD recovery. This intensive approach, including prazosin, allows the patient to adopt and improve coping skills during the course of treatment, leading to a global improvement of all PTSD symptoms. Some research suggest that prazosin, through the activation of alpha-1 adrenergic receptors in the prelimbic cortex, may influence the reconsolidation of fear memory, leading to a better discrimination between safe and threatening stimuli (42).

Prazosin showed similar effects in both sexes; however, we are not powered to determine if this observed lack of difference in treatment response related to sex or to the type of trauma is accurate because sexual trauma was the majority of trauma exposures within our female population. A lower response to treatment was observed in patients with repetitive sexual abuse; therefore, knowing the cumulative effect of multiple or chronic exposure could be associated with a poorer response to prazosin; however, a larger trial would be necessary to evaluate this hypothesis.

The apparent effects of prazosin in this study are consistent with the suspected pathophysiology of PTSD that includes the sympathetic nervous system, hypothalamic-pituitary-adrenal axis and the catecholaminergic system (43). The α_1_-adrenergic receptor, blocked by prazosin, mediates several physiological effects of epinephrine and norepinephrine (44). Norepinephrine is a primary mediator of the human stress response and its role in the pathophysiology of PTSD has been studied. Specifically, patients with PTSD have shown increased activation of norepinephrine (45) and are hyper-responsive to a variety of stimuli (46). Higher cerebrospinal fluid concentration of norepinephrine has been demonstrated in patients with PTSD compared to controls. The concentration of norepinephrine has been correlated with the severity of the PTSD symptoms (47). Many PTSD symptoms are related to heightened adrenergic function: arousal, sensibility to novel stimuli, selective attention and vigilance, enhanced memory encoding for arousing and aversive events and in subsequent re-experiencing symptoms such as intrusive memories and nightmares (45). In the pediatric population, limited data suggest that the locus ceruleus, the sympathetic nervous system and the catecholamine system are dysregulated in traumatized youth who may suffer from PTSD and depressive symptoms (48). In a pilot study with sexually abused girls, baseline 24-hour urinary catecholamine concentrations were elevated (25). Elevated plasma norepinephrine levels were found in children who developed PTSD after a motor vehicle accident compared to children who did not exhibit PTSD in the same context (24).

In the study, one patient experienced a tolerance issue (hypotension). The treatment was not discontinued due to significant clinical improvement but instead the dose was decreased to 1 mg/day. Prazosin appears to be a safe treatment in pediatric PTSD. The current data about the tolerance in youth with PTSD show that the adverse events reported are consistent with the well characterized side-effect profile of prazosin (35). In adult populations, prazosin showed no significant effect on systolic or diastolic BP (49). Moreover, no rebound hypertension is reported at discontinuation (35), unlike with alpha-2 agonists such as clonidine and guanfacine (50).

With the exception of case reports, a systematic review (34) and a retrospective study from Keeshin et al. (35), the current literature regarding the use of prazosin in pediatric population mainly concerns chronic hypertension and bladder neck dysfunction. For hypertension, alpha-blockers such as prazosin are Food and Drug Administration (FDA) approved but are not considered first-line drugs for hypertension in accepted guidelines, due to the better efficacy of new drugs (latest FDA, report 2014). However, expert reports do not exclude its use in the pediatric population (51).

The safety data regarding α-blockers in the pediatric population mainly concern the treatment of neurogenic bladder (NB). Importantly, to date, there is no report of serious adverse events associated with α-blocker treatment (off label prescribed) in children. Kroll et al. did not report any serious adverse events regarding α-blocker therapy (including prazosin) in children with NB (52). In a study of 208 children with bladder dysfunctions treated with α-blockers (including prazosin), Golębiewski et al. found minor adverse effects in only five children (53). In another study of 55 patients treated with doxazosin (a drug similar to prazosin), minor adverse effects were observed in two children, and in another group of 16 boys there were no significant adverse effects (54, 55). Changes of systolic and diastolic BP were negligible. Finally, a small study of 14 children identified one boy who experienced drowsiness and a decrease in BP and therefore could not complete the study (56).

It is important to note that prazosin, similar to other antiadrenergic agents, does not induce metabolic adverse effects. This is in contrast to the commonly observed side effect profile of antipsychotics in youth. Considering the lack of efficacy data among youth with PTSD treated with antipsychotics, antiadrenergic agents may be an appropriate alternative to use rather than a trial of an antipsychotic in pediatric PTSD.

The results of this study should be interpreted in the context of its limitations. First, the number of patients was low despite the large proportion of PTSD patients (12%) among all our inpatients admitted between January 2016 and December 2017. This could be explained by the fact that all of them were acutely ill patients, recruited in a university teaching hospital that may be particularly enriched in subjects with a more severe form of PTSD.

Secondly, our study was based on data retrospectively analyzed and used during the course of one year. The clinical evaluation, including answers to the scales, could have been influenced by the fact that both the clinician and the patient were aware and informed of the use of prazosin.

Third, prazosin was mainly prescribed in association with another previous psychotropic treatment rather than alone. Even though the dosage of previously initiated medications was maintained and not modified during the prazosin trial, these treatments could also have led to improvement of PTSD symptoms, even though the lack of efficacy on PTSD symptoms from these previous treatments also supported their admission in our unit and the use of prazosin. Fifteen patients received a psychotropic treatment in addition to prazosin including: fluoxetine, sertraline, aripiprazole, quetiapine, and carbamazepine. Quetiapine and carbamazepine might have had an impact due their association with improvement in symptoms in cases of pediatric PTSD (20, 21). For the other medications, it is difficult to discriminate the effect on PTSD symptoms from psychiatric comorbidities (anxiety, depression). Furthermore, the multidisciplinary and integrative approach is likely to contribute to the patient’s improvement. Additionally, PTSD scores may have improved simply from being hospitalized and/or receiving other types of interventions.

Fourth, the duration of our study was only 4 weeks. The efficacy of prazosin was evaluated to study its use in acutely ill youth. Even though our data support the association with improvement in intrusive symptoms, our data cannot illuminate long term treatment outcomes. In an outpatient cohort, Keeshin et al. study (35), followed patients for a longer period of time (10.2 ± 8.1 weeks). In that study, persistent improvement associated with prazosin was noted. A recent randomized trial involving military veterans who had chronic PTSD studied the efficacy of prazosin in a long-term follow up. No significant differences were reported at 26 weeks in nightmares and sleep disorders between the prazosin group and the placebo group (33). However, a randomized controlled study on young soldiers was significantly positive up to 14 weeks of treatment (31). We consider prazosin as a promising approach to symptomatic treatment that provides a rapid improvement of PTSD symptoms, which may assist in the transition to evidence based psychotherapies. However, the optimal duration of treatment with prazosin needs to be defined.

The fifth limitation is the dose-response effect. In our study, the dose of prazosin varied from 1 to 3 mg. This dose is lower than in adult population trials and in Keeshin et al. study (up to 15 mg). Two patients in our cohort might have benefited from an increase to 3 mg or higher as they were still experiencing sleep difficulties but parents or caregivers did not allow a dose increase. We observed that 1 mg of prazosin was associated with a significant improvement in symptoms; however, we could not test for dose-response effects because of clear limitations in our design. There is a need for randomized trials to establish the optimal prazosin dose in pediatric PTSD.

Sixth, we only assessed the patient report for PTSD symptoms. Parents report was not systematically gathered serially during hospitalizations. Previous studies have shown empirically that single-respondent information may result in a large underestimate of PTSD severity (57, 58).

Finally, the UCLA-PSTD-RI is the current gold standard for the assessment of PTSD in pediatric population; however, we acknowledge that the UCLA-PSTD-RI is not appropriate for all cases, especially for patients with intellectual or developmental delay, who might have difficulties to understand the questions of the interview. Thus, two children were not included in our study even though they received prazosin because of the severity of their symptoms. However, they also showed a positive clinical response to prazosin.

The strengths of this study include i) the first and largest sample of patients systematically and prospectively assessed regarding the use of prazosin in pediatric PTSD, ii) new data supporting previous case series, iii) the large amount of data assessed prospectively for each patient, iv) an extensive and systematic monitoring of adverse events to evaluate the safety of prazosin in youth with PTSD, v) the use of the UCLA-PSTD-RI to systematically assess and monitor symptoms. Finally, this encouraging case series and its results led to design an ongoing French national multicenter randomized controlled trial comparing prazosin versus placebo in youth with PTSD, sponsored and endorsed by the French Health Government (*PHRC National 2019*, *PHRC-19-0067*, *FINESS*: *760780239*, https://solidarites-sante.gouv.fr/systeme-de-sante-et-medico-social/recherche-et-innovation/l-innovation-et-la-recherche-clinique/appels-a-projets/programmes-recherche *).*

## Conclusion

To our knowledge, this report is the first study offering prospective clinical data regarding the use of prazosin for clinical purpose in a large pediatric PTSD sample. Our study findings have several clinical implications for youth suffering from PTSD. First, our results demonstrate that prazosin was associated with improvement of sleep disturbances, intrusive symptoms and other symptoms of pediatric PTSD. Second, prazosin was well-tolerated except for one child who experienced hypotension. Third, prazosin may be an appropriate therapeutic option for youth with PTSD. Fourth, prazosin was associated with improvement in coping skills regarding threatening stimuli and therefore its efficacy could be enhanced if an integrative therapeutic approach is associated with its use. However, these promising results need to be supported and confirmed with randomized controlled trials.

## Data Availability Statement

The raw data supporting the conclusions of this article will be made available by the authors, without undue reservation.

## Ethics Statement

The studies involving human participants were reviewed and approved by Comité d’Ethique pour la Recherche sur Données Existantes et/ou hors loi Jardé (CERDE), CHU de Rouen. Written informed consent to participate in this study was provided by the participants’ legal guardian/next of kin.

## Author Contributions 

VF contributed conception, design of the study and acquisition of data, wrote the first draft of the manuscript, and reviewed the draft. MS contributed conception and design of the study and wrote the first draft of the manuscript. BC analyzed of data for the work and wrote the first draft of the manuscript. AM, J-MG, and BK wrote sections of the manuscript. PG revised it critically for important intellectual content. All authors contributed to the article and approved the submitted version.

## Conflict of Interest

The authors declare that the research was conducted in the absence of any commercial or financial relationships that could be construed as a potential conflict of interest.
