# Supplementary material for: Use of Prazosin for Pediatric Post-Traumatic Stress Disorder With Nightmares and/or Sleep Disorder: Case Series of 18 Patients Prospectively Assessed
Source: Front Psychiatry. 2020 Jul 22;11:724. doi: 10.3389/fpsyt.2020.00724 (PMC7388897; doi:10.3389/fpsyt.2020.00724)
Supplement: Supplementary file 1 [file Table_1.docx]

**Supplementary table 1: mean of each cluster week after week**

| Variable | Baseline, mean | 1 week, mean | 2 weeks, mean | 3 weeks, mean | 4 weeks, mean | Variance  (improvement pourcentage) |
| --- | --- | --- | --- | --- | --- | --- |
| UCLA-PSTD-RI global score | 56.44 | 45.06 | 35.56 | 24.00 | 18.5 | -37.94  (67.2) |
| Intrusion  (cluster B) | 16.44 | 12.60 | 8.61 | 4.39 | 2.61 | -13.83  (84.1) |
| Avoidance  (cluster C) | 4.89 | 3.39 | 2.33 | 1.06 | 0.78 | -4.11  (84.1) |
| Negative cognition and mood (cluster D) | 16.67 | 13.78 | 11.83 | 8.72 | 7.39 | - 9.28  (55.7) |
| Arousal  (cluster E) | 18.67 | 15.39 | 12.50 | 9.56 | 7.67 | -11.0  (58.9) |
| Dissociative symptoms  (cluster A) | 2.89 | 2.56 | 1.67 | 0.72 | 0.44 | -2.4  (84.6) |
| Sleep score | 6.44 | 5.06 | 3.44 | 2.44 | 1.33 | 5.11  (79.3) |

**Supplementary Table 2: clinical assessment of the tolerance and specific prazosin dose achieved for each patient**

| Patients | Systolic blood pressure  Baseline  (mmHg) | Systolic blood pressure  Endpoint  (mmHg) | Diastolic blood pressure  Baseline  (mmHg) | Diastolic blood pressure  Endpoint  (mmHg) | Heart rate  Baseline  (bpm) | Heart rate  Endpoint  (bpm) | Adverse effects | Prazosin dose  (mg/day) |
| --- | --- | --- | --- | --- | --- | --- | --- | --- |
| 1 | 104 | 95 | 66 | 60 | 77 | 76 | Hypotension (after increasing at 2 mg/day)  BP: 65/46 mmHg  HR: 130 bpm | 1 |
| 2 | 130 | 110 | 90 | 80 | 101 | 72 | None | 3 |
| 3 | 120 | 120 | 80 | 70 | 67 | 90 | None | 3 |
| 4 | 110 | 105 | 75 | 60 | 96 | 96 | None | 2 |
| 5 | 125 | 120 | 80 | 80 | 92 | 97 | None | 3 |
| 6 | 90 | 110 | 60 | 80 | 90 | 100 | None | 1 |
| 7 | 115 | 100 | 80 | 60 | 101 | 93 | None | 2 |
| 8 | 120 | 120 | 80 | 80 | 80 | 80 | None | 3 |
| 9 | 100 | 105 | 60 | 65 | 72 | 92 | None | 2 |
| 10 | 96 | 108 | 79 | 80 | 93 | 99 | None | 2 |
| 11 | 120 | 120 | 80 | 80 | 80 | 80 | None | 2 |
| 12 | 107 | 117 | 70 | 82 | 102 | 114 | None | 2 |
| 13 | 120 | 121 | 80 | 75 | 70 | 72 | None | 2 |
| 14 | 105 | 110 | 65 | 70 | 98 | 93 | None | 2 |
| 15 | 120 | 105 | 70 | 75 | 100 | 100 | None | 2 |
| 16 | 110 | 100 | 60 | 70 | 91 | 88 | None | 2 |
| 17 | 116 | 120 | 85 | 80 | 83 | 88 | None | 3 |
| 18 | 107 | 110 | 64 | 70 | 81 | 81 | None | 2 |

**Supplementary table 3: difference of baseline scores depending on the type of trauma and the sex**

|  | **General** | **After sexual abuse** | **After familial violence** | **p-value** | **In male** | **In female** | **p-value** |
| --- | --- | --- | --- | --- | --- | --- | --- |
| **UCLA-A** | 2.89 +/- 1.64 | 3.08 +/- 1.80 | 2.40 +/- 1.14 | 1 | 2.62 +/- 0.92 | 3.10 +/- 2.08 | 0.31 |
| **UCLA-B** | 16.44 +/- 3.60 | 16.62 +/- 3.45 | 16.00 +/- 4.36 | 1 | 16.75 +/- 3.73 | 16.20 +/- 3.68 | 1 |
| **UCLA-C** | 4.89 +/- 2.08 | 4.62 +/- 2.10 | 5.60 +/- 2.07 | 0.61 | 4.87 +/- 2.30 | 4.90 +/- 2.03 | 0.66 |
| **UCLA-D** | 16.67 +/- 5.41 | 17.54 +/- 5.22 | 14.40 +/- 5.81 | 0.31 | 15.50 +/- 5.98 | 17.60 +/- 5.04 | 0.19 |
| **UCLA-E** | 18.67 +/- 4.46 | 19.77 +/- 3.90 | 15.80 +/- 4.97 | 0.29 | 17.88 +/- 5.06 | 19.30 +/- 4.08 | 0.64 |
| **UCLA-Total** | 56.44 +/- 12.78 | 58.62 +/- 12.24 | 50.80 +/- 13.77 | 0.29 | 54.50 +/- 13.51 | 58.00 +/- 12.68 | 0.64 |
| **CGI-IS** | 5.28 +/- 0.90 | 5.31 +/- 1.03 | 5.20 +/- 0.48 | 0.62 | 5.63 +/- 0.92 | 5.00 +/- 0.82 | 1 |
